# Socioeconomic inequalities in health-related quality of life between men and women, 5 years after a coronary angiography

**DOI:** 10.1186/s12955-016-0570-z

**Published:** 2016-12-03

**Authors:** Anastase Tchicaya, Nathalie Lorentz

**Affiliations:** LISER- Luxembourg Institute of Socio-Economic Research, LISER, 11 Porte des Sciences, L4366 Esch-Sur-Alzette, Luxembourg

**Keywords:** Cardiovascular diseases, Health-related quality of life, Gender, WHOQOL-BREF, Socioeconomic status, Luxembourg

## Abstract

**Background:**

The aim of this study is to measure gender differences in health-related quality of life (HRQOL) among men and women patients with cardiovascular diseases (CVD), and to assess the impact of socioeconomic factors on HRQOL between men and women, 5 years after a coronary angiography.

**Methods:**

The study included 1,289 out of 4,391 patients who had undergone an angiography in the National Institute for Cardiac Surgery and Interventional Cardiology, Luxembourg in 2008/2009. Four indicators of the WHOQOL-BREF questionnaire (Self-rated health, Quality of life, Physical health, and Psychological health) were used in this study as interest variables. To assess the socioeconomic inequalities in HRQOL between men and women, general linear models were constructed for every indicator, with educational level and living conditions as predictors, and demographic variables, cardiovascular risk factors, and cardiovascular events as covariates.

**Results:**

Women were older than men (71.5 versus 68.1, *p* <0.0001) and less likely to be married. HRQOL was significantly different between men and women despite the fact they had the same socioeconomic status. The average score for overall health was 3.7/5 for men versus 3.5/5 for women; similarly, the life quality score was 3.8/5 for men versus 3.6/5 for women. Education level and living conditions were associated with lower HRQOL scores in men and women.

**Conclusion:**

The findings showed that women have lower HRQOL than men regarding self-rated health, quality of life, and the WHOQOL-BREF physical and psychological domains 5 years after a coronary angiography. Socioeconomic inequalities affect HRQOL, and their influence was similar in both men and women. Socioeconomic inequalities in HRQOL in women and men with CVD are strong 5 years after a coronary angiography. Taking into account differences in gender and socioeconomic status in intervention strategies to substantially reduce the differences observed between women and men could help improve the effectiveness of secondary prevention.

## Background

Cardiovascular diseases (CVD) are the leading cause of death or morbidity worldwide [1, 2]. These are more common in men than in women, but in the past, they have often been diagnosed at a late age [2–4]. Indeed, in the USA, for example, the lifetime risk of developing CVD after age 40 was 49% in men and 32% in women [3]. These disparities persist between men and women in cardiovascular health and care, and specifically in the delivery and outcomes of coronary revascularization therapy [3]. For example, unadjusted mortality and complication rates remain significantly higher in women treated with percutaneous coronary intervention (PCI) than in men [3]. The impact of risk factors is higher among women than men [4, 5]. The quality of life related to health is a good predictor of the disease and its evolution [6, 7]. Quality of life is a multidimensional indicator that integrates physical, psychological, emotional, and social factors [8]. Health-related quality of life (HRQOL) represents the functional effect of an illness and its consequent therapy, as perceived by the individual [9]. Many studies have shown that health-related quality of life in women after a coronary event or with cardiovascular disease is lower than in men [2, 10–15]. For example, women with CVD are more likely than men to face continuous demands in the home or family environment and are also more likely to neglect their health care needs [16, 17]. Women are particularly challenged by resuming their traditional household activities soon after discharge from the hospital [13]. However, time spent on household activities is not necessarily a predictor of worse quality of life in women [18]. In a recent study, women reported lower HRQOL scores than men in both the ‘EuroQoL self-rated health grade and RAND-36’ [15].

Several studies have also shown that socioeconomic and demographic factors had a significant influence on HRQOL both in the general population and in people already suffering from CVD [8, 19–21]. Several studies comparing HRQOL between female and male patients with CVD have failed to take into account socioeconomic status in their analyses. Indeed, it is known that patients with low socioeconomic status experience a significantly lower HRQOL compared to patients with high socioeconomic status [21, 22]. In Italy, for example, Chiara et al. [23] found that less-educated patients were more likely to have worse cardiovascular health compared with medium-highly-educated patients. The presence of a high global cardiovascular risk in patients with lower levels of education might be attributed to an insufficient understanding by the patient of the importance of proper management of cardiovascular risk factors [23], and/or to a poor knowledge of major cardiovascular risk factors [24].

The aim of this study is to measure health-related quality of life among men and women patients with CVD, 5 years after a coronary angiography in the National Institute of Cardiac Surgery and Interventional Cardiology (INCCI) in Luxembourg, and to evaluate the impact of socioeconomic status on health-related quality of life between men and women. To the best of our knowledge, a comparable approach has not been reported before.

## Methods

Data derived from a follow-up study of 4391 patients admitted for a coronary angiography in INCCI from 1 January 2008 to 31 December 2009 who participated in the research project ‘State of health and its determinants -ESANDE’ [24]. Patients were contacted again 5 years later (August 2013-April 2014) [21]. The follow-up survey was conducted by mail with a self-administered questionnaire. Patients were asked to complete a written informed consent form after being informed of the research objectives. A total of 1837 files were accessed (among whom there were 548 deaths), representing a response rate of 42% compared to the patient population of the base year 2008–2009. Excluding deaths, only information on 1289 patients could be used in the follow-up study. Due to the lack of information concerning HRQOL and living conditions at the baseline, this study was performed with a cross-sectional perspective.

### Health-related quality of life

Health-related quality of life was measured according to the WHOQOL-BREF Questionnaire [21, 25]. The WHOQOL questionnaire is a generic instrument to assess quality of life, defined as the ‘individual’s perceptions of their position in life in the context of the values and culture systems in which they live and in relation to their goals, expectations, standards, and problems’ [25].

Two of the four domains of the WHOQOL-BREF questionnaire were selected for this study, namely, the physical health and psychological health domains. The scores of each domain were transformed into a scale from 0 to 100 points, with higher scores indicating better quality of life related to health.

Two other health-related quality of life items of the WHOQOL-BREF questionnaire representing global perceptions of quality of life and assessment of general health were used.

### Socioeconomic variables

Two socioeconomic variables were used: education level and living conditions. The educational level was defined as the highest diploma level according to the International Standard Classification of Education and subdivided into three levels: primary, secondary, and tertiary education [26].

Living conditions were defined using the following question: ‘considering the monthly resources of your household, how would you say that they allow you to live?’ Response items ‘very hardly’ and ‘hardly’ were grouped into ‘hardly’ and response items ‘easily’ and ‘very easily’ into ‘easily’.

### Demographic variables

Age, sex, and marital status were used as demographic variables in the analysis. Age was subdivided into four levels: less than 54 years old, 55–64 years old, 65–74 years old, and 75 years old and more. Marital status was defined as a dichotomous variable: ‘married’ or ‘other’.

### Cardiovascular risk factors

Five cardiovascular risk factors were retained for this study based on their high prevalence among individuals who suffered from CVD: weight (change), diabetes, cholesterol, hypertension, and smoking status. Weight change was measured with the question ‘In the past 12 months, did you: lose weight, gain weight, or was your weight stable?’ Based on the responses, a self-reported, three-category weight change variable was constructed that included patients who reported weight gain, weight loss, and stable weight. For diabetes, hypertension, and cholesterol, dichotomous variables were used: ‘yes’ if the patient reported high glycaemia/high blood pressure/high cholesterol levels and ‘no’ if the patient did not report suffering from either of these risk factors. The use of tobacco was assessed using three categories: ‘current smoker’, ‘ex-smoker’, and ‘never-smoker’.

### Cardiovascular events

Ischemic heart disease, acute myocardial infarction, and stable angina pectoris were the most observed cardiovascular events among the patients undergoing a coronary angiography in 2008-2009. Both events were defined as dichotomous variables: yes, if the patient had the pathology (or a health problem related to it), and no, if the patient did not have the pathology [21].

### Statistical analyses

Two types of analysis were performed: descriptive and multivariate analyses. Descriptive analysis used Chi-square tests to compare the baseline distribution of patients’ characteristics between men and women, and HRQOL according to gender and socioeconomic status (i.e. level of education and living conditions) in the follow-up data.

Multivariate analysis was performed using general linear model (GLM) procedures.

As a consequence, least square multiple regression analysis was used to examine the relation between HRQOL of patients (the domains ‘physical health’ and ‘psychological health’, self-rated health, and quality of life) as outcome variables and socioeconomic status (education and living conditions). This relation was assessed after adjusting for socio-demographic variables (age, marital status), cardiovascular risk factors (use of tobacco, weight change, diabetes, high blood pressure, and high cholesterol level), and cardiovascular events (diagnosis). This analysis was performed using the GLM procedure of SAS 9.4 (SAS Institute Inc., USA).

## Results

The distribution of socioeconomic characteristics, cardiovascular risk factors, and cardiovascular events in patients by gender are presented in Table 1.

**Table 1:** Characteristics of the patients

	Men	Women	*p*-value
N	911 (70.7%)	378 (29.3%)	
Age	68.1 (+-10.6)	71.5 (+-12.3)	<0.0001
Less than 54	11.1	10.1	<0.0001
55-64	23.2	15.9	
65-74	36.8	28.8	
75 and more	29.0	45.2	
Marital status			
Married	80.4	52.9	<0.0001
Other	19.6	47.1	
Education level			
Primary	32.0	46.2	<0.0001
Secondary	49.6	47.2	
Tertiary	18.3	6.6	
Living conditions			
Easy living conditions	85.7	82.0	0.1106
Difficult living conditions	14.3	18.0	
Weight change			
Weight loss	18.5	19.9	0.0214
Weight gain	17.3	23.4	
No change	64.2	56.7	
Diabetes			
Yes	29.9	28.5	0.6473
No	70.1	71.5	
Hypertension			
Yes	39.3	49.4	0.0019
No	60.8	50.6	
Cholesterol			
Yes	45.6	51.1	0.0940
No	54.4	48.9	
Smoking status			
Yes, current	11.0	7.8	<0.0001
No, ex-smokers	62.0	33.4	
No, never	27.0	58.8	
Angina Pectoris			
Yes	47.3	45.2	0.4971
No	52.7	54.8	
Acute myocardial infarction			
Yes	10.2	7.7	0.1567
No	89.8	92.3	
Ischemic heart disease			
Yes	13.1	10.3	0.1713
No	86.9	89.7	

Source: Monitoring and Dynamics of health status through the Risk Factors for Cardiovascular disease (MDYNRFC) Survey, 2013/2014

Our sample consists of 70.7% men and 29.3% women. Women were older than men (71.5 versus 68.1, *p* <0.0001) and less likely to be married. They also differed from men by their lower level of education (only 6.6% of women had a higher level of education versus 18.3% for men). They had higher levels of hypertension (49.4%) and cholesterol than men (39.3%) and were more likely to have gained weight during the 12 months preceding the survey than men (23.4% vs. 17.3%, *p* <0.0214). However, women were less likely to smoke or to be ex-smokers (respectively 7.8% vs. 11.0%, *p* <0.0001; 33.4% vs. 62.0%, *p* <0.0001). More than half of them had never smoked, while among men only 27% had never smoked.

HRQOL was significantly different between men and women, despite the fact that they had the same socioeconomic status (Table 2). The average score for overall health was 3.7/5 for men versus 3.5/5 for women; similarly, the life quality score was 3.8/5 for men versus 3.6/5 for women.

**Table 2:** Health-related quality of life according to gender and socioeconomic factors

	Men		Women		*p*-value
	*N*	Mean (SD)	*N*	Mean (SD)	
Self-rated health	868	3.73 (0.75)	353	3.48 (0.80)	<0.0001
Quality of life	904	3.78 (0.72)	374	3.60 (0.79)	<0.0001
WHOQOL-BREF physical domain	867	62.80 (17.86)	336	53.90 (19.70)	<0.0001
WHOQOL-BREF psychological domain	869	70.97 (16.12)	337	65.61 (17.69)	<0.0001
Education level					
Primary					
Self-rated health	270	3.56 (0.73)	160	3.38 (0.81)	0.0196
Quality of life	289	3.62 (0.72)	173	3.54 (0.79)	0.2765
WHOQOL-BREF physical domain	271	57.01 (17.72)	150	49.58 (19.92)	<0.0001
WHOQOL-BREF psychological domain	272	67.07 (16.58)	151	63.57 (18.33)	0.0457
Secondary					
Self-rated health	434	3.74 (0.74)	170	3.55 (0.80)	0.0054
Quality of life	447	3.79 (0.71)	175	3.59 (0.79)	0.0022
WHOQOL-BREF physical domain	432	64.38 (17.05)	161	55.72 (18.38)	<0.0001
WHOQOL-BREF psychological domain	433	72.01 (15.81)	161	66.79 (17.08)	0.0005
Tertiary					
Self-rated health	158	3.94 (0.74)	22	3.59 (0.67)	0.0363
Quality of life	163	4.06 (0.64)	25	4.04 (0.61)	0.8752
WHOQOL-BREF physical domain	159	68.15 (17.51)	24	68.85 (18.83)	0.8568
WHOQOL-BREF psychological domain	159	74.84 (14.98)	24	70.56 (17.00)	0.2014
Living conditions					
Difficult living conditions					
Self-rated health	119	3.34 (0.69)	59	3.07 (0.93)	0.0519
Quality of life	126	3.29 (0.76)	61	3.18 (0.81)	0.3836
WHOQOL-BREF physical domain	122	51.27 (18.10)	53	41.42 (19.52)	0.0015
WHOQOL-BREF psychological domain	121	60.41 (18.15)	54	53.32 (17.41)	0.0167
Easy living conditions					
Self-rated health	719	3.80 (0.73)	263	3.56 (0.74)	<0.0001
Quality of life	749	3.87 (0.68)	281	3.68 (0.75)	0.0002
WHOQOL-BREF physical domain	721	64.89 (16.91)	258	56.38 (18.95)	<0.0001
WHOQOL-BREF psychological domain	724	72.82 (14.95)	258	68.17 (16.61)	<0.0001

Source: Monitoring and Dynamics of health status through the Risk Factors for Cardiovascular disease (MDYNRFC) Survey, 2013/2014

Quality of life scores were statistically higher in men than in women. Participants scored lowest in the domain of physical health; men (62.8/100 on average) scored higher than women (53.9/100 on average). Regarding the psychological domain, the score was 71.0/100 for men and 65.6/100 for women.

The significant differences between men and women also persisted depending on the socioeconomic characteristics of patients, except for some quality of life indicators related to health (Table 2). For equivalent levels of education, the HRQOL scores in men were statistically higher than those of women, except for the indicators ‘quality of life’ for primary (*p* = 0.2765) and higher tertiary (*p* = 0.8752) levels of education, and ‘WHOQOL Physical’ and ‘WHOQOL psychological’ for higher levels of education (*p* = 0.8568 and *p* = 0.2014, respectively) only. For equivalent living conditions, men had higher HRQOL scores than women, except for the indicators ‘self-rated health’ and ‘quality of life’ among those with difficult living conditions (*p* = 0.0519 and 0.3836, respectively).

Indeed, regardless of socioeconomic status, perceived health was greater in men than in women, with the exception of those declaring difficult living conditions. The score of quality of life did not differ between men and women except for those with a secondary education, and those whose conditions were easy. In the physical and psychological health domains, women's scores were statistically lower than those of men regardless of socioeconomic status, except for those with a higher level of education.

The association between HRQOL and socioeconomic factors is presented in Table 3.

**Table 3:** Association between health-related quality of life and gender and socioeconomic factors (adjusted for age, marital status, weight change, diabetes, high cholesterol, hypertension, smoking status, and diagnosis)

	Self-rated health	Quality of life	WHOQOL-BREF physical domain	WHOQOL-BREF psychological domain
	Estimate (Standard error)	Estimate (Standard error)	Estimate (Standard error)	Estimate (Standard error)
Gender				
Men	ref.	ref.	ref.	ref.
Women	-0.24 (0.06)^a^	-0.11 (0.06)^c^	-7.77 (1.36)^a^	-4.42 (1.26)^b^
Education level				
Primary	-0.31 (0.07)^a^	-0.36 (0.08)^a^	-9.12 (1.07)^a^	-5.95 (1.58)^b^
Secondary	-0.12 (0.07)	-0.27 (0.06)^a^	-3.95 (1.56)^c^	-2.37 (1.44)
Tertiary	ref.	ref.	ref.	ref.
Living conditions				
Difficult living conditions	-0.40 (0.07)^a^	-0.48 (0.07)^a^	-11.51 (1.62)^a^	-11.09 (1.51)^a^
Easy living conditions	ref.	ref.	ref.	ref.

^a^<0.0001, ^b^<0.01, ^c^<0.05

Source: Monitoring and Dynamics of health status through the Risk Factors for Cardiovascular disease (MDYNRFC) Survey, 2013/2014

Adjusted for age, marital status, cardiovascular events, risk factors, and socioeconomic position, women had lower self-rated health (estimate: -0.24, standard error: 0.06), as well as quality of life (estimate: -0.11, standard error: 0.06), including the physical health domain (estimate: -7.77, standard error: 1.36) and the psychological health domain (estimate: -4.42, standard error: 1.26). Education level and living conditions were also associated with each indicator of quality of life. Low socioeconomic position was associated with the lowest health-related quality of life.

In Table 4, the associations between HRQOL and socioeconomic factors are presented according to gender for each HRQOL indicator.

**Table 4:** Association between health-related quality of life and socioeconomic factors according to gender (adjusted for age, marital status, weight change, diabetes, high cholesterol, hypertension, smoking status, and diagnosis)

	Men	Women
	Estimate (Standard error)	Estimate (Standard error)
Self-rated Health		
Education Level		
Primary	-0.32 (0.08)^a^	-0.20 (0.20)
Secondary	-0.15 (0.07)^c^	0.02 (0.19)
Tertiary	ref.	ref.
Living conditions		
Difficult living conditions	-0.36 (0.08)^a^	-0.57 (0.15)^a^
Easy leaving conditions	ref.	ref.
Quality of life		
Education level		
Primary	-0.35 (0.07)^a^	-0.43 (0.18)^c^
Secondary	-0.26 (0.07)^b^	-0.39 (0.18)^c^
Tertiary	ref.	ref.
Living conditions		
Difficult living conditions	-0.53 (0.08)^a^	-0.33 (0.14)^c^
Easy leaving conditions	ref.	ref.
WHOQOL-BREF physical domain		
Education level		
Primary	-7.17 (1.87)^a^	-17.23 (4.56)^b^
Secondary	-2.60 (1.67)	-10.69 (4.35)^c^
Tertiary	ref.	ref.
Living conditions		
Difficult living conditions	-11.27 (1.83)^a^	-12.22 (3.58)^b^
Easy leaving conditions	ref.	ref.
WHOQOL-BREF psychological domain		
Education level		
Primary	-5.87 (1.74)^b^	-6.21 (4.18)
Secondary	-2.72 (1.55)	-1.50 (4.00)
Tertiary	ref.	ref.
Living conditions		
Difficult living conditions	-10.14 (1.71)^a^	-15.20 (3.28)^a^
Easy leaving conditions	ref.	ref.

^a^<0.0001, ^b^<0.01, ^c^<0.05

Source: Monitoring and Dynamics of health status through the Risk Factors for Cardiovascular disease (MDYNRFC) Survey, 2013/2014

Self-rated health was associated with education level and living conditions in men. In women, only living conditions had a significant association, but the impact of living conditions on self-rated health was more important for women than for men (estimate in women: -0.57, estimate in men: -0.36).

The general quality of life indicator was associated with education level and living conditions in both men and women. However, the impact of education level was more important in women than in men. Indeed, estimates were higher in women with primary and secondary education (respectively -0.43 and -0.39) than in men with primary and secondary education (respectively -0.35 and -0.26). However, impact of living conditions was more important in men than in women (-0.53 against -0.33).

Lower scores in the physical health domain were associated with low education level and difficult living conditions in both men and women. The impact of difficult living conditions was the same in men and women (respectively -11.24 and -12.22), but the impact of primary education level was more important for women than for men (-17.23 in women, -7.17 in men).

Lower scores in the psychological health domain in men were associated with low education level and difficult living conditions. In women, only difficult living conditions were significantly linked with the psychological health domain, but with a more important impact than in men (-15.20 versus -10.14).

Despite a higher percentage of women with the lowest educational level, this circumstance does not seem to affect some aspects of the quality of life of these women, such as self-rated health or the WHOQOL-psychological domain.

## Discussion

This study confirms that women have lower self-rated health, lower quality of life, lower physical health, and lower psychological health than men. This is consistent with previous research literature [16, 27–30].

For example, Emery et al. [16] showed that the quality of life related to physical health is worse among women than men with heart disease. After a 12-month follow-up, physical HRQOL had increased among both men and women, but women still had a lower quality of life than men [16]. Hartman et al. [28] showed that after PCI, women had worse HRQOL scores in the short and medium term than men (1 and 3 years) but in the long term (10 years) these differences disappeared [28]. Baumann et al. [29] showed that life satisfaction was lower in female than in male patients 5 years after a review of coronary angiography [21, 29]. This difference could be explained by the fact that women are increasingly confronted with continuing demands at home, and neglect their health needs [16, 31].

Moreover, quality of life could be more strongly associated with social support among women than men [16, 31]. Ford et al. [27] also speculated that the differences in quality of life between men and women could be due to the differences in treatment of the disease, rather than the severity of illness, as well as presence of comorbidities, use of social media, and psychosocial factors [27]. Other authors propose that women have a higher prevalence of depressive symptoms, and limitations in physical and social activities that increase stress and frustration [32]. Most of these studies have shown the existence of differential effects of CVD on HRQOL between men and women. These differences are multidimensional in origin. This study sought to go beyond the male/female dichotomy to analyse the influence of socioeconomic factors on HRQOL among men and women.

This study not only shows that low socioeconomic position is associated with low quality of life, but also that socioeconomic inequalities are more important in women than in men, except for self-rated health or the WHOQOL-psychological domain.

Indeed, Ghasemi et al. [33] showed that women with coronary heart disease had a better quality of life when they had a higher level of education or a good income compared to women with a low education level or a low income.

The Women’s Ischemia Syndrome Evaluation study showed that low socioeconomic status was the best predictor of mortality and morbidity in women with symptoms of myocardial ischemia [8]. Economically and socially disadvantaged women have more symptoms, have a poor quality of life, and decreased survival rates compared to women with higher socioeconomic status [8]. In Sweden, in a study of hospitalized women, László et al. [34] found an inverse relationship between personal income and adverse outcomes. The explanation of socioeconomic inequalities in health can be formulated from two assumptions: i) the health selection hypothesis (health selection) and ii) the reverse causality hypothesis (reverse causation) that determine the social position health [35, 36].

Compared to men, the risk of CVD in women is increased to a greater extent by some traditional risk factors (e.g. diabetes, hypertension, hypercholesterolaemia, obesity), and socioeconomic and psychosocial factors continue to have a high impact on CVD in women [37].

The gender differences in health-related quality of life could be affected by comorbid depression that is present more often among women [13, 37]. Indeed, comorbid depression could function as a mediator between gender and HRQOL. Many studies have shown the effects of depressive symptoms on HRQOL in patients with coronary artery disease [38] and patients undergoing coronary artery bypass graft [13, 39, 40].

It has been found that structural and psycho-social determinants globally tend to be more important to women's health, while behavioural determinants tend to be more important to men’s health [37, 41].

Furthermore, gender differences in CVD symptoms, management, and outcome have been observed. It has been noted that women underestimate their risk of CVD because the general public still perceives CVD as primarily a health problem for men [37, 42]. However, despite the increase in CVD awareness among women, outcomes in women are still worse than in men. The management of CVD in men and women is ‘obviously different’, and these differences are partly due to gender biases in favour of men [37].

### Limitations and strengths

This study, based on monitoring data from a cohort of patients five years after coronary angiography, has some limitations. There is a risk of possible bias due to the current composition of respondents compared to the base sample due to non-response. Another limitation is the fact that HRQOL was not measured at baseline at the time of examination of coronary angiography. It was not possible to measure changes in HRQOL in patients during the study interval as well as the evolution of socioeconomic inequalities in HRQOL. The difficulty of obtaining data on individual patients limits the analysis of the complexity of gender differences related to health. Some other clinical variables that have been reported in other studies, for example in Dueñas et al. [31], and that affect the HRQOL of both sexes have not been included, such as angina frequency or rehospitalisation. Comorbidity was another clinical variable that was not considered in this study. It could explain, at least to some extent, the differences obtained, for example in comorbid depression [37]. However, the follow-up survey was not able to collect information concerning the presence of other pathologies.

Furthermore, the small size of the sample limits our ability to conduct stratified analyses by diagnosis or type of cardiovascular event and gender.

Despite these limitations, this study provides evidence on the association between socioeconomic status (educational level, living conditions) and HRQOL scores among CVD patients 5 years after a coronary angiography, evidence that may be useful to policy-makers, and specifically be useful in clinical settings. It was clearly shown that HRQOL scores for women were significantly lower than those of men. Similarly, among patients of the same sex, those with low socioeconomic status were more likely to have a HRQOL low score compared with those with a higher socioeconomic status. Nevertheless, in women, the lowest educational level did not affect HRQOL more than the highest educational level, specifically HRQOL indicators such as self-rated health and psychological health.

## Conclusions

Our findings showed that women have lower HRQOL than men regarding self-rated health, quality of life, and the WHOQOL-BREF physical and psychological domains 5 years after a coronary angiography. Socioeconomic inequalities affect HRQOL, and their influence was similar in both men and women. Socioeconomic inequalities in HRQOL in women and men with CVD are strong. HRQOL differences are also pervasively present even for those in comparable socioeconomic positions. Taking into account differences in gender and socioeconomic status may be necessary when planning intervention strategies (treatment and/or rehabilitation) to substantially reduce the differences observed between women and men, as well as between disadvantaged and advantaged patients within each sex, to improve the effectiveness of secondary prevention.

## Abbreviations

CVD: Cardiovascular diseases
GLM: General linear model
HRQOL: Health-related quality of life
INCCI: National institute of cardiac surgery and interventional cardiology
